# Research Progress Concerning a Novel Intraocular Lens for the Prevention of Posterior Capsular Opacification

**DOI:** 10.3390/pharmaceutics14071343

**Published:** 2022-06-25

**Authors:** Yidong Zhang, Chengshou Zhang, Silong Chen, Jianghua Hu, Lifang Shen, Yibo Yu

**Affiliations:** 1 Eye Center, The Second Affiliated Hospital, School of Medicine, Zhejiang University, Hangzhou 310058, China; yidongzhang@zju.edu.cn (Y.Z.); zhangchengshou@zju.edu.cn (C.Z.); cshelo@zju.edu.cn (S.C.); laurel_hu@163.com (J.H.); y214180453@163.com (L.S.); 2 Jiande Branch, The Second Affiliated Hospital, School of Medicine, Zhejiang University, Hangzhou 310058, China

**Keywords:** posterior capsular opacification, intraocular lens, surface modification, drug delivery, photothermal therapy, photodynamic therapy, micro-pattern, anti-biofouling

## Abstract

Posterior capsular opacification (PCO) is the most common complication resulting from cataract surgery and limits the long-term postoperative visual outcome. Using Nd:YAG laser-assisted posterior capsulotomy for the clinical treatment of symptomatic PCO increases the risks of complications, such as glaucoma, retinal diseases, uveitis, and intraocular lens (IOL) pitting. Therefore, finding how to prevent PCO development is the subject of active investigations. As a replacement organ, the IOL is implanted into the lens capsule after cataract surgery, but it is also associated with the occurrence of PCO. Using IOL as a medium for PCO prophylaxis is a more facile and efficient method that has demonstrated various clinical application prospects. Thus, scientists have conducted a lot of research on new intraocular lens fabrication methods, such as optimizing IOL materials and design, and IOL surface modification (including plasma/ultraviolet/ozone treatment, chemical grafting, drug loading, coating modification, and layer-by-layer self-assembly methods). This paper summarizes the research progress for different types of intraocular lenses prepared by different surface modifications, including anti-biofouling IOLs, enhanced-adhesion IOLs, micro-patterned IOLs, photothermal IOLs, photodynamic IOLs, and drug-loading IOLs. These modified intraocular lenses inhibit PCO development by reducing the residual intraoperative lens epithelial cells or by regulating the cellular behavior of lens epithelial cells. In the future, more works are needed to improve the biosecurity and therapeutic efficacy of these modified IOLs.

## 1. Introduction

Cataracts, the leading cause of blindness globally, is a disease that results in color changes or opacification of a transparent ocular lens and leads to a series of visual defects [1]. Nowadays, researchers consider cataract lens removal surgery combined with intraocular lens (IOL) implantation as the only effective treatment for vision-limiting cataracts [2,3]. Although cataract phacoemulsification surgery is mature, post-surgical complications, such as posterior capsular opacification (PCO), limit the long-term postoperative visual outcomes [4]. PCO incidence ranges from 20% to 40% within five years after cataract surgery in adults, while in children, the incidence is as high as 100% [5,6]. Using Nd:YAG laser-assisted posterior capsulotomy, the clinical management of symptomatic PCO, may lead to several risks, such as glaucoma, retinal diseases, uveitis, and intraocular lens (IOL) pitting [7,8,9]. Therefore, PCO prevention is still the subject of active investigation.

### 1.1. Pathophysiology of PCO

**Figure 1:**
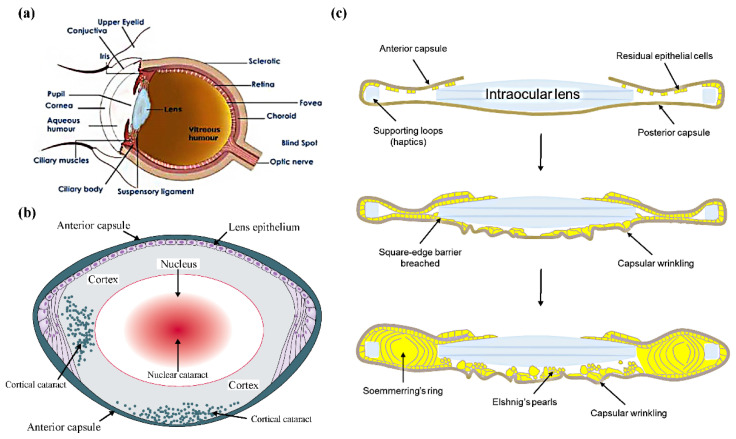
(**a**) The structure of a human eye. Adapted with permission from Ref. [10]. Copyright 2015 Elsevier. (**b**) The structure of a human lens. Adapted with permission from Ref. [1]. Copyright 2017 Elsevier. (**c**) The PCO development following cataract surgery. Adapted with permission from Ref. [5]. Copyright 2021 Elsevier.

The lens sits behind the iris inside the eye and is attached to the ciliary body through zonules [10] (Figure 1a). Histologically, it is composed of the lens capsule (including the anterior capsule, the equatorial region, the acellular posterior capsule), fibers, and single-layer lens epithelium [1]. Lens fibers are transformed from the lens epithelium and compacted from the periphery to the center. Thus, older lens fibers make up the core (nucleus) of the lens, and newly formed lens fibers make up the outer layer of the lens, the cortex (Figure 1b).

The pathophysiology of PCO is still not fully elucidated. Through the surgery of the extracapsular cataract extraction (including the phacoemulsification surgery), the nucleus and cortex were removed, and the lens capsule retains for the IOL implantation [2,3]. Despite the initial success of cataract surgery, there are always residual lens epithelial cells (LECs), as well as the postoperative inflammation, in the capsular bag (Figure 1c). Since cataract surgery initiates a wound-healing response, these living LECs start to proliferate and migrate over all available surfaces, including regions below or outer of the anterior capsule; IOL surfaces; and of primary importance the previously acellular posterior capsule, ultimately encroaching on the visual axis [4,5]. Furthermore, LECs undergo transdifferentiation into myofibroblasts and the process of epithelial–mesenchymal transformation (EMT). In addition, aberrant differentiation is considered to result in swollen globular cells, shown as the structure of Elschnig’s pearls. Additionally, the structure of Soemmerring’s ring is formed by lens fiber differentiation in the peripheral capsular bag (outside the IOLs). Thus, matrix deposition, capsular wrinkling, increased cell aggregation, and Elschnig’s pearls collectively cause significant visual disruption [11]. Moreover, changes in growth factors (such as transforming growth factor, fibroblast growth factor, epidermal growth factor, and insulin-like growth factor), cell adhesion molecules (such as intracellular adhesion molecule-1, various integrin ligands, and CD44), extracellular matrix (ECM) components (such as fibronectin, vitronectin, and collagen), other signaling molecules, and signal pathways play a critical role in regulating residual LEC functions and behaviors [5].

### 1.2. The Role of IOLs in PCO Prevention

According to the pathogenesis of PCO, the current research on PCO prevention has focused on reducing the residue of LECs or regulating their cellular behaviors. The cellular behaviors of LECs and the development of PCO are closely related to IOL biocompatibility. Amon et al. [12] have proposed for the first time that the IOL biocompatibility can be divided into the uveal and capsular biocompatibility. The uveal biocompatibility refers to the foreign body inflammatory response of uveal tissue to the IOL. The disruption of the blood–aqueous humor barrier during cataract surgery causes the influx of proteins and cells into the anterior chamber and their adhesion to the IOL surface. This protein adhesion plays a dramatic role in accumulating other cells, and the inflammatory cells convert into macrophages and giant cells, inducing a foreign body response. Capsular biocompatibility mainly refers to the opacification of the anterior and posterior capsule because of the proliferation and migration of LECs or the growth of LECs on the anterior surface of the IOL [13].

The IOL biocompatibility is influenced by several factors, including the IOL material, the optical edge design, and surface properties [14]. A more hydrophilic IOL surface is generally considered for better uveal biocompatibility, while a more hydrophobic IOL surface means better capsular biocompatibility [14]. The hydrophilic IOL surface reduces the adhesion of proteins and cells, which results in an alleviated postoperative inflammatory response, but it provides a suitable interface for the proliferation and migration of LECs. Numerous studies have demonstrated that constructing the hydrophilic anti-biofouling coatings on the IOL surface could reduce PCO incidence by inhibiting the adhesion, proliferation, or migration of LECs on the IOL surface [15,16,17,18]. Conversely, PCO has also been regarded as a unique form of an inflammatory response in which inflammatory cells (such as macrophages and giant cells) secrete cytokines and, in turn, regulate cellular behaviors of LECs, leading to severe PCO [13,19,20]. Therefore, the uveal and capsular biocompatibility of the IOL is not separate but closely related properties, and the effect of IOL surface hydrophobicity on PCO formation should also be treated critically. Improving the IOL biocompatibility through surface modification is a potential method for PCO prevention [14,21].

As the replacement organ is directly implanted in the eye, the IOL can serve as a medium to reduce postoperative residual LECs or regulate their behaviors through physical or biochemical means. This is a more facile and efficient method with clinical application prospects for PCO prophylaxis. For example, the IOL can be a drug delivery device that delivers anti-inflammatory, cytotoxic, and antiproliferative drugs that prevent PCO. This has the advantage of improving drugs and prolonging the time of action of drugs [22,23]. A significant benefit of drug-loaded IOLs is ensuring continuous drug delivery, better drug bioavailability, and patient compliance.

In addition to optimizing cataract surgery techniques and the intraoperative irrigation of drug solution in the capsule, scientists have conducted lots of research on using the intraocular lens to prevent PCO formation. They have focused on optimizing the IOL materials, design, and modifying surface properties (such as plasma/ultraviolet/ozone treatment, chemical grafting, drug loading, layer-by-layer self-assembly methods, and coating modification) [22,23,24,25].

**Figure 2:**
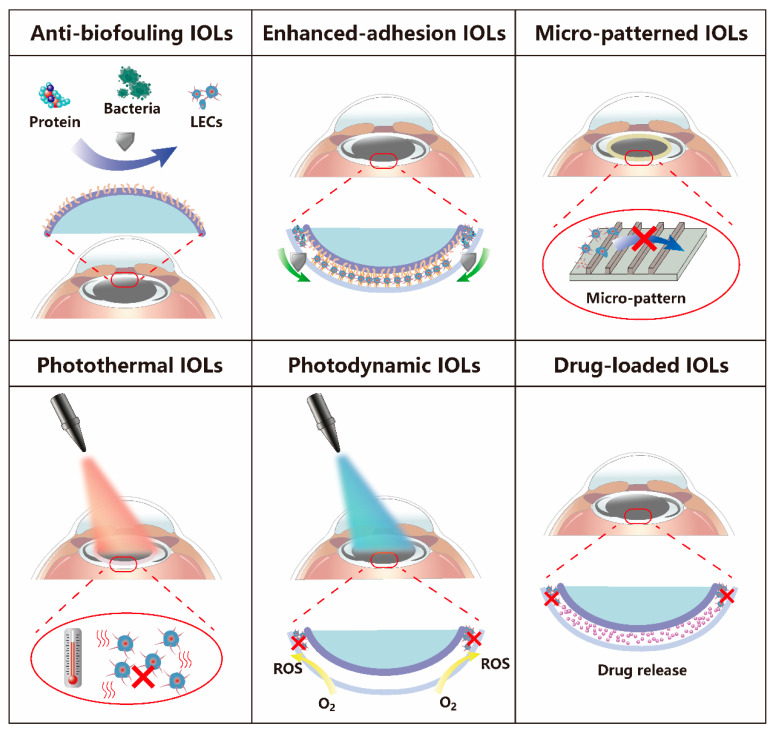
Schematic illustration of the main types of IOLs used for PCO prophylaxis.

This article mainly places greater focus on the different intraocular lenses prepared by IOL surface modification for PCO prevention, including anti-biofouling IOLs, enhanced-adhesion IOLs, micro-patterned IOLs, photothermal IOLs, photodynamic IOLs, and drug-loading IOLs. We summarize their brief mechanisms for PCO prevention in Figure 2.

## 2. The Main Types of IOLs Used for PCO Prophylaxis

### 2.1. Anti-Biofouling IOLs

Biofouling means the undesired adsorption and adhesion of biomolecules, cells, or microorganisms, and the formation of microbial films on material surfaces [26]. Since these biofouling components would induce inflammatory responses and potentially cause infection and/or immunologic rejection, the property of anti-biofouling is significantly relevant to the durability and security of medical applications, for example, medical implants, contact lenses, catheters, hemodialyzers, biosensors, and respirators [26,27,28,29]. Antifouling polymers or coatings modified onto these medical devices change their surface characteristics and thus improve biocompatibility and the performance in resisting biofouling.

The fabrication of anti-biofouling IOLs aims to alleviate the inflammatory response and decrease the LEC number on the IOL surface. This is performed by reducing the adhesion of proteins, bacteria, and cells, resulting in the inhibition of PCO formation. Previous studies have induced hydrophilic groups into IOL surfaces, such as heparin, vinyl pyrrolidone, and α-allyl glucoside, and enhanced the anti-biofouling and anti-inflammation ability of IOLs [21]. Nevertheless, in a clinical trial, heparin-surface-modified (HSM) hydrophilic acrylic IOLs and hydrophobic acrylic IOLs were implanted in cataract patients for 12 months of investigation [30]. In the uncomplicated cataract patients, the respective PCO areas and PCO severity scores were 6.12% and 0.081 in the HSM IOLs group and 5.91% and 0.075 in the control group, respectively. There was no statistically significant difference in the PCO area or the PCO severity score between the two IOLs, demonstrating no superiority of HSM IOLs in preventing PCO. Thus, these anti-fouling IOLs require more investigation to improve the PCO prevention effect before the final clinical application. The reviewed literature of anti-fouling IOLs for PCO prophylaxis with in vivo results is summarized in Table 1.

Poly(ethylene glycol) (PEG), a neutral and hydrophilic polymer with low PEG–water interface energy and no immunogenicity, is a commonly used anti-biofouling material. Lee et al. [31] applied PEG molecules for the surface modification of acrylic IOL with a square edge design. The PEG-IOLs were implanted in the rabbit models and found to reduce PCO severity at 36 weeks significantly. Scanning electron microscopy showed more LECs tightly adhered to the IOL surface without PEG modification. However, PEG-IOLs did not substantially inhibit PCO formation after seven weeks. Xu et al. [32] immobilized the hydrophilic PEG onto the IOL surface via a plasma-aided chemical grafting procedure without influencing the optical properties. This PEG-modified IOL had good biocompatibility in the rabbit model and exerted a PCO inhibition effect for more than four months. Subsequently, the research group fabricated the hydrophilic poly(poly(ethylene glycol) methacrylate) (PPEGMA) brush on the IOL surface using reversible addition fragmentation chain-transfer (RAFT) technology, which was an even more stable modification of the PEG. The anti-biofouling coating was sufficient to inhibit LEC adhesion and proliferation on the IOL, thereby reducing the incidence of PCO [17].

The compound 2-Methacryloyloxyethyl Phosphorylcholine (MPC) is an amphoteric material with excellent hydrophilicity, biocompatibility, and a specific bionic structure that provides the artificial cell membrane interface. These features allow for various applications in biological implants, tissue engineering, and drug delivery systems [33]. MPC forms a membrane-like structure and traps water molecules on the acrylic or silicone IOL, resulting in significantly improved surface hydrophilicity and anti-adhesion of proteins, cells, and bacteria [34,35,36].

Han et al. [37] prepared amphoteric MPC brushes on the surface of IOL acrylic acid by the bottom-up grafting method, and this alleviated the PCO severity after implanting MPC-modified IOL into rabbit eyes. Similarly, Tan et al. [38] synthesized the hydrophilic copolymer P (MPC-MAA) from the negatively charged hydrophilic methyl acrylic acid (MAA) and MPC, and they covalently grafted the copolymer onto the hydrophobic acrylic IOL surface after ammonia plasma treatment. However, the MPC-MAA modification could inhibit postoperative inflammation and anterior capsular opacification (ACO) rather than PCO. In another study, a polymer containing the amphoteric betaine group was used by Wang et al. [39] for surface modification because of its good biocompatibility, lubricity, and anti-biofouling properties in the hydration state. The hydrophilic poly(sulfobethine methacrylate) (PSBRMA) brush coating was prepared on the IOL using the RAFT method. This decreased LEC adhesion and proliferation in vitro and significantly reduced the turbidity of the posterior capsule in vivo.

The natural polysaccharide is extensively used in the surface modification of biomaterials, such as hyaluronic acid (HA) and chitosan (CHI). HA is a negatively-charged polysaccharide, naturally existing in the vitreous body, joint, and skin. However, CHI is the only cationic natural polysaccharide [40,41]. Considering the advantages of the hydration property of polysaccharides, Lin et al. [42] constructed a hydrogel-like polyelectrolyte multilayer coating composed of HA and CHI components on the surface of the silicone IOL using the electrostatic layer-by-layer (LBL) self-assembly method. The anti-biofouling coating of HA/CHI inhibited LEC adhesion and proliferation. For the in vivo experiments, modified IOLs prevented the central PCO (3 mm diameter), but the severity of peripheral PCO and Soemmerring’s ring was not significantly different between the control and modified IOL groups.

**Figure 3:**
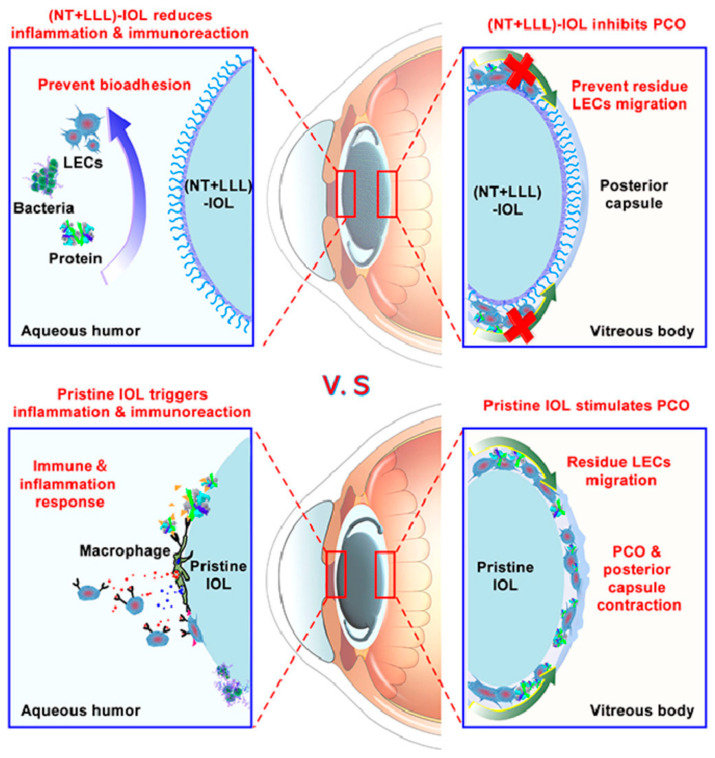
Schematic illustration of the (NT + LLL)-IOL with advantages of anti-bio-adhesion and PCO inhibition, compared to the pristine IOL. Adapted with permission from Ref. [43]. Copyright 2021 Elsevier.

The modified IOLs mentioned above were rarely resistant to biofouling while maintaining tight contact with the posterior capsule. Thus, Wu et al. [43] fabricated a new type of IOL with a hydrophobic anti-biofouling coating (Figure 3). After functionalizing the nanomorphology of the hydrophobic IOL surface and covalently conjugating it with a “liquid-like” polydimethylsiloxane brush, the resulting (NT + LLL)-IOL exhibited strong resistance to biological fouling, including proteins, cells, and bacteria, because of the low surface energy of the modified surface. The hydrophobic surface of the (NT + LLL)-IOL had a better attachment to the posterior capsule, thus preventing residual LEC migration. Therefore, the (NT + LLL)-IOL implantation alleviated the intraocular inflammation response postoperatively and PCO formation. Despite having a liquid-like layered coating, the (NT + LLL)-IOL still had advantages of optical transparency, good biocompatibility, and mechanical robustness.

**Table 1:** Summary of reviewed literature of anti-biofouling IOLs for PCO prophylaxis with in vivo results.

Composition	Main Fabrication Method	IOL Type	Observation	Prophylaxis Effect	Ref.
PEG	oxygen plasma-aided activation and grafting polymerization	acrylic IOL (SA60AT, Alcon)	eight weeks in rabbit model	alleviate PCO formation for six weeks, but had no effect afterwards	[31]
PPEGMA	oxygen and argon plasma-aided activation and grafting polymerization	acrylic IOL (SN60WF, Alcon)	four months in rabbit model	alleviate PCO formation	[32]
PPEGMA	oxygen plasma-aided activation and RAFT grafting polymerization	acrylic IOL (SN60WF, Alcon)	six months in rabbit model	alleviate PCO formation	[17]
MPC	RAFT grafting polymerization	acrylic IOL (SN60WF, Alcon)	one month in rabbit model	alleviate PCO formation	[37]
MPC/MAA	ammonia plasma-aided activation and grafting polymerization	acrylic IOL (Eyegood Medical Tech.)	eight weeks in rabbit model	alleviate anterior capsular opacification formation, but did not alleviate PCO formation	[38]
PSBMA	RAFT grafting polymerization	acrylic IOL (66Vision Tech.)	six months in rabbit model	alleviate PCO formation	[39]
HA/CHI	layer-by-layer assembly	acrylic IOL (Alcon)	one month in rabbit model	alleviate CPCO formation not PPCO	[42]
PDMS	oxygen plasma-aided activation and chemical vapor deposition	acrylic IOL (Eyebright Medical Tech.)	two months in rabbit model	alleviate PCO formation	[43]

### 2.2. Enhanced-Adhesion IOLs

According to the “sandwich” theory proposed by Linnola et al. [44,45,46,47], the IOL surface with biological adhesion properties allows the LECs to adhere to the IOL, forming the three-tier structure of the IOL, monolayer LECs, and posterior capsule membrane. The sealed sandwich structure prevents further LEC proliferation and migration, thereby reducing the incidence of PCO. The monolayer LEC’s proliferation probably only slightly influences the contrast sensitivity and not transparency of posterior capsules. In addition to LECs, the proteins that make up the extracellular matrix, including fibronectin, vimentin, laminin, and collagen IV, can play a role in this adhesion mode [45,46].

Thus, the molecular basis of relatively lower PCO incidences in hydrophobic IOL than hydrophilic IOL has been speculated by protein adsorption behaviors [48,49]. The hydrophobic surface is considered more bio-sticky to the posterior capsule than hydrophilic one via adsorbing more proteins and via the adsorbed adhesion protein-induced cell layer [44,45,46]. On the other hand, the similarly shaped hydrophobic acrylic IOLs with stronger adhesive force were shown to inhibit LEC migration and PCO more than IOLs with weaker adhesive force [50]. Arjun et al. [51] proved fibronectin adsorption of simulated posterior capsules significantly reduced simulated LEC infiltration between hydrophobic acrylic IOLs and the posterior capsules by increasing adhesion forces compared with fibronectin-free controls. Additionally, LECs slow down the rate of the differentiation once they are well attached [52].

Previous studies have shown that the ultraviolet/ozone (UV/O_3_) or argon plasma-treated IOL surfaces could enhance LEC and protein adhesion, thereby solidifying the binding between the IOL and posterior capsule membrane [53]. Both UV/O_3_ treatment and argon plasma treatment increased nitrogen substituents and functional OH and COOH functional groups, thus improve surface characteristics (e.g., wettability and adhesion). Additionally, the COOH groups are highly adhesive to protein fibronectin [54]. The post-treated IOLs significantly inhibited PCO formation in comparison to control IOLs, and the UV/O_3_ treatment was more effective than the argon plasma treatment [53]. Additionally, UV/O_3_ treatment causes little damage to the IOL surface, whereas argon plasma may promote surface deterioration through an etching effect. Farukhi et al. [55] used UV/O_3_ treatment to modify the posterior surface of IOLs, which achieved similar PCO prevention effects in in vivo animal experiments.

The RGD peptide (Arg-Gly-Asp sequence) is the primary functional motif of fibronectin that promotes cell adhesion [45,46,56]. The RGD modification method has been widely studied for promoting cell adhesion onto material surfaces in cancer therapy and diagnosis [57]. The biomimetic strategy of RGD peptide-grafting on the IOL surface has been demonstrated superiority in enhancing the LEC adhesion without inducing the EMT process of LECs in vitro experiments [58]. The surface modification did not produce a significant change in the optical and mechanical properties of IOLs. The IOL with RGD peptide-functionalization has the potential to reconstruct the sandwich structure of the IOL-LEC capsule biomaterial. However, the effect of PCO prophylaxis should be further confirmed by in vivo experiments. On the other hand, macrophages, fibroblastic cells, and other types of cells will recognize the RGD peptide via their surficial integrins and attach to RGD-functionalized surfaces non-discriminatorily [56,59]. The RGD-based strategy lacks biological specificity and thereby has no direct regulation effect on other cell processes, for example, cell differentiation.

### 2.3. Micro-Patterned IOLs

The edge design of IOLs is closely related to cell migration and PCO formation. Nishi et al. [60] first demonstrated that the sharp-edge design of IOLs could decrease PCO incidence in clinical practice by inhibiting LEC migration from the IOL edge to the central optical axis [61,62].

In fact, cell migration is based on the interaction of focal adhesions (proteins embedded in the cell membrane) with biomaterial interfaces. Additionally, the unique micro-surface or micro-patterned topography could direct specific biological behaviors, such as cell migration in the eyes, by regulating the placement of focal adhesions [63,64]. Magin et al. [65] reported that silicone protective membrane (PM) with different Sharklet micro-patterns could inhibit LEC migration on its surface in an in vitro PCO model compared to the PM without micro-patterns. Additionally, the micro-patterns protruding from the surface reduced migration more than the recessed features. Thus, Kramer et al. [66] implanted the Sharklet micro-patterned PM in the capsule after cataract surgery and then embedded the IOL in the PM device, which resulted in PCO inhibition. Subsequently, the research group directly incorporated a Sharklet micro-patterned membrane on the posterior surface of the IOL peripheral rim to effectively alleviate PCO severity [67].

**Figure 4:**
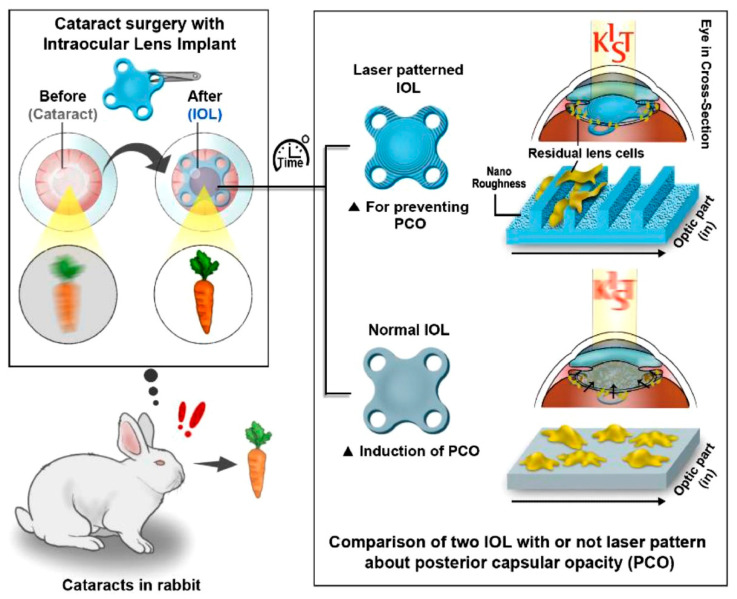
Schematic illustration of nano-textured micro-patterning on intraocular lens to suppress posterior capsular opacification by regulating cell behavior, such as adhesion, migration, and proliferation. Adapted with permission from Ref. [73]. Copyright 2020 Elsevier.

The femtosecond laser (FL) processing of materials, known as laser ablation, is a facile and efficient technique for creating desired micro-patterned interfaces that control focal cell adhesion, migration, differentiation, and other behaviors [68,69,70,71,72]. For example, the FS-ablated electrospun scaffolds facilitate endothelial cell ingrowth and increase the M2 macrophage and overall cell infiltration [69]. Additionally, FL-patterned silicon substrates with micro-cone arrays were shown to work as a potential and valuable platform for patterning neurons into artificial networks [71]. Another study also investigated the effect of periodic nano-textured patterns on LEC behaviors. Here, the researchers innovatively prepared the micro-patterned IOL samples of poly(HEMA) using femtosecond laser (FL) microfabrication (Figure 4). These groove/ridge patterns inhibited the migration of the single and collective LECs in a width-dependent mode. The groove with a size comparable to the size of cells exhibited the most significant inhibition effect [73]. Additionally, the proliferative rates of LECs were also slightly decreased on the nano-textured patterned surface. Experiments in vivo demonstrated that the implantation of commercially available IOLs modified by the FL ablation did not induce intraocular inflammation, but effectively reduced PCO severity for eight weeks. Furthermore, the FL induced ablation uses ultrashort laser pulses (~100 fs) to minimize thermal stress and collateral damage to materials, such as metal, ceramic, silicon, and polymer, resulting in stable and reproducible patterning processing. This facile and precise strategy for FL microfabrication is free of chemical treatment and allows for potential application in various biomaterials for regulating cell behaviors in biomedical devices and implants.

### 2.4. Photothermal IOLs

As a non-invasive treatment method, near-infrared (NIR) photothermal therapy (PTT) has been widely studied and used in the treatment of many diseases [74,75,76]. Nanomaterials with photothermal conversion properties can have a therapeutic role when converting light energy into heat energy under the irradiation of near-infrared light (700–1400 nm wavelength). Compared with drug treatment, PTT treatment minimizes collateral damage to other tissues through controllable nanomaterial distribution and laser irradiation. Carbon-based nanocomposites (such as graphene derivatives and carbon nanotubes), metal nanomaterials (such as Au nanorods), polymers, and other nanocomposites (such as polydopamine) are often used in the development of PTT because of their excellent photothermal conversion properties [77].

**Figure 5:**
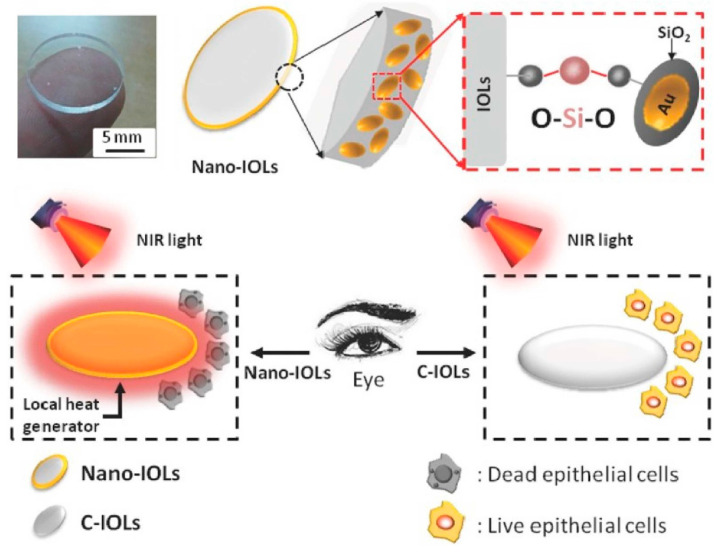
The schematic illustration of nano-IOLs with nanostructured Au@SiO_2_ outer rim for prevention of posterior capsule opacification. Adapted with permission from Ref. [76]. Copyright 2017 John Wiley and Sons.

Recent literature of photothermal IOLs for PCO prophylaxis with in vivo results is summarized in Table 2. Lin et al. [76] took the lead in applying PTT to remove residual LECs in the eye and prevent PCO (Figure 5). SiO_2_-coated Au nanorods (Au@SiO_2_) were integrated into the edge of the IOL using the activation immersion method to obtain nanostructured photothermal ring-modified IOLs. The photothermal IOL has excellent biocompatibility and optical properties, and has a regional restrictive photothermal effect. This allows it to accurately eliminate LECs in vivo and in vitro without affecting other tissues in the eye, thus inhibiting the progress of PCO.

Because of the high cost, complex synthesis, and potential long-term cytotoxicity of gold nanomaterials, Xu et al. [78] used polydopamine (PDA) as the photothermal modification material for IOLs because it is easier to obtain and prepare and has better biocompatibility. The PDA coating was deposited on the surface of the rim annulus of the IOL by a CuSO_4_/H_2_O_2_-triggered PDA rapid deposition technique. Compared with normal IOLs, the modified IOLs could play an influential role in sterilization and PCO prevention under NIR light irradiation. Reduced graphene oxide (rGO)-based nanocomposites have been widely used in PPT for advantages of pronounced photothermal conversion effect, facile synthesis, good biocompatibility, and low cost [79,80,81]. A previous study proved that repeated rGO exposure did not cause eye toxicity in mouse models [82]^.^ Thus, our team fabricated a polyethyleneimine (PEI)/reduced graphene oxide (rGO) thin film coatings on IOL surface by controllable layer-by-layer self-assembly. The rGO@IOL implant with postoperative NIR irradiation showed promise for clinical applications in PCO prophylaxis [83]. Moreover, carboxylated CuInS/ZnS nano-quantum dots without toxic heavy metals have also been used to develop photothermal IOLs [84].

**Figure 6:**
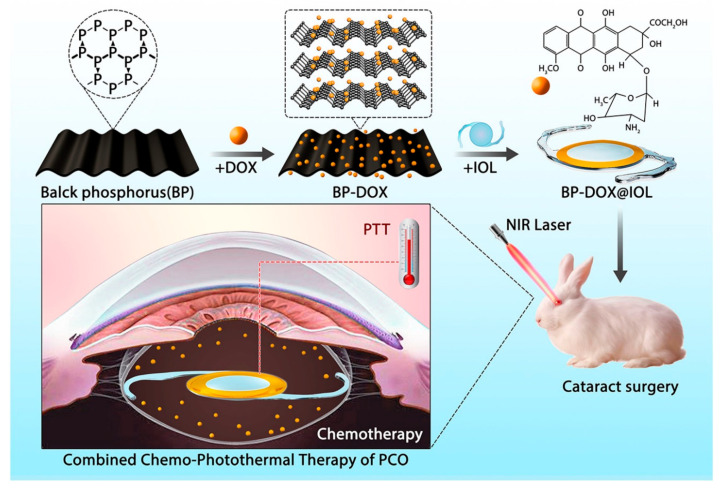
The schematic illustration of BP-based drug delivery system for synergistic chemo-photothermal therapy of PCO. Adapted with permission from Ref. [85]. Copyright 2021 Elsevier.

To enhance the efficiency of photothermal IOLs, Mao et al. [85] introduced a combined photothermal therapy and chemotherapy strategy for PCO prevention (Figure 6). BP-DOX@IOL was prepared by assembling the antiproliferative drug doxorubicin (DOX)-loaded black phosphorus (BP) nanosheets onto the non-optical section of the commercial IOL. The NIR light irradiation triggered the release of DOX loaded by BP-DOX@IOL and led to the area-limited heating of the IOL-modified area through the photothermal conversion effect, which jointly destroyed LECs efficiently. Although the BP-DOX@IOL inhibited the progress of PCO to a certain extent after implantation in the eye, the superposition of postoperative near-infrared light treatment achieved a more excellent PCO prevention effect.

**Table 2:** Summary of reviewed literature of photothermal and photodynamic IOLs for PCO prophylaxis with in vivo results.

Composition	Mechanism	Main Fabrication Method	IOL Type	Observation	Irradiation Protocol In Vivo	Ref.
Au nanorods/SiO_2_	PTT	oxygen plasma-aided activation and immersion	commercial acrylic IOL	thirty days in rabbit model	808 nm, 3.3 W/cm^2^, 10 min; once a week	[76]
PDA/PEI	PTT	CuSO_4_/H_2_O_2_-triggered rapid deposition	acrylic IOL (66Vision Tech.)	four weeks in rabbit model	808 nm, 0.3 W/cm^2^, 10 min; at day 1, 3, 5, 7, 14, 21, and 28	[78]
rGO/PEI	PTT	plasma-aided activation and layer-by-layer self-assembly	acrylic IOL (66Vision Tech.)	four weeks in rabbit model	808 nm, 2.5 W/cm^2^, 10 min; three times in the first week, twice in the second week, and once a week in subsequent weeks	[84]
BP/DOX	PTT and chemotherapy	facial activation and immersion	acrylic IOL (Eyebright Medical Tech.)	four weeks in rabbit model	808 nm, 1 W/cm^2^, 3 min; once a week from the second week	[85]
ICG/PLGA	PDT	facial activation, electrostatic attraction and immersion	commercial IOL	eight weeks in rabbit model	785 nm, 120 mW/cm^2^, 10 min; every day for one month	[86]
α-CD-Ce-6/PPEGMA	PDT	RAFT technology and supramolecular self-assembly	acrylic IOL (66Vision Tech.)	two months in rabbit model	660 nm, 2.4 W/cm^2^, 2 min; once a day in the first week	[87]
Ce-6/PDA	PDT	self-polymerization	acrylic IOL (66Vision Tech.)	four weeks in rabbit model	660 nm, 2.4 W/cm^2^, 2 min; once a day for two weeks	[88]

### 2.5. Photodynamic IOLs

Photodynamic therapy (PDT) uses photosensitizers (PSs) under the irradiation of specific wavelengths of light to convert oxygen into reactive oxygen species (ROS), thereby inducing cell apoptosis. PDT therapy has been widely used to treat diseases with abnormal cell proliferation, such as cancer [89]. Similar to PTT treatment, PDT can minimize collateral damage to other tissues through controlled illumination and the distribution of photosensitive materials.

Previous PDT regimens that used photosensitizer solution directly perfuse the capsular bag followed by light exposure, which may induce potential ocular toxicity [90,91]. Therefore, modifying photosensitizer materials on the surface of IOL is a feasible approach (summarized in Table 2). Indocyanine green (ICG), a non-toxic and water-soluble dye, is a commonly used reagent in ophthalmic clinical applications and a photosensitizer material. Zhang et al. [86] prepared the photodynamic IOLs by adsorbing ICG molecules with positively-charged IOLs and sealing them with polylactic-co-glycolic acid (PLGA). Because of the presence of ICG, the light transmittance of the assembled ICG-IOL decreases. However, after implantation into the eye, with the gradual degradation of the PLGA, the water-soluble ICG is released from the IOL, and the light transmittance of the IOL can be restored. ICG-IOL combined with laser irradiation (785 nm) effectively reduced LEC activity and inhibited LEC proliferation and migration. In vivo implantation experiments showed that the ICG-IOL-based PDT system could significantly prevent the occurrence of PCO and had good biocompatibility.

**Figure 7:**
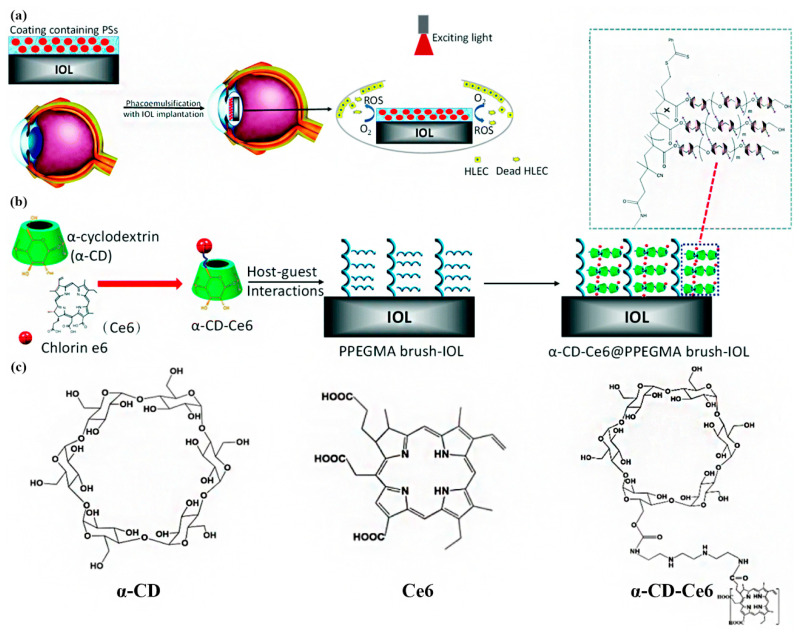
(**a**) Schematic illustration of an IOL with PS-containing coating and the process of PDT. (**b**) Schematic of the surface coating structure of the IOL. (**c**) Chemical structure of α-CD, Ce6, and α-CD-Ce6. Adapted with permission from Ref. [87]. Copyright 2021 Royal Society of Chemistry.

Tang et al. [87] used chlorin e6 (Ce6) as a model photosensitizer and grafted it onto α-cyclodextrin (α-CD) molecules to synthesize α-CD-Ce6 (Figure 7). Subsequently, utilizing the host–guest interaction between PPEGMA and α-CD, a PPEGMA-α-CD-Ce6 coating with photodynamic therapy function was constructed on the surface of the previously PPEGMA brush-modified IOL [17,87]. In vitro experiments confirmed that the photodynamic IOL could generate a large amount of ROS under 660 nm laser irradiation, effectively inducing the apoptosis of LECs and inhibiting PCO development in vivo. In other research, this group attached Ce6 to the IOL surface when fabricating highly viscous PDA coatings via the rapid self-polymerization of dopamine. It also proved that the PDT coating based on the Ce6 photosensitizer could eliminate residual LECs significantly and prevent PCO formation [88].

### 2.6. Drug-Loaded IOLs

As drug delivery devices for handling postoperative cataract complications, IOLs can improve drug bioavailability, ensure sustained drug release, and enhance patient compliance, with broad clinical application prospects [22]. Drug-loaded IOLs can be constructed using direct drug solution soaking, supercritical impregnation, drug reservoir attachment, and surface modification. To prevent the PCO, drug-loaded IOLs can transport anti-inflammatory, antineoplastic, anti-cell migration, or anti-EMT drugs into the capsular bag [22,23]. Thus, the reviewed literature of drug-loaded IOLs for PCO prophylaxis with in vivo results is summarized in Table 3.

It has been suggested that the inflammatory response after cataract surgery induces LEC proliferation, migration, EMT, and other cell behaviors, which may accelerate the process of PCO [92]. The cyclooxygenase-2 (COX-2) level in LECs is associated with PCO development, and it is often elevated by injury or inflammatory cytokines [93]. Nonsteroidal anti-inflammatory drugs (NSAIDs) exert anti-inflammatory effects by inhibiting COX-2. After finding that the selective NSAIDs Celecoxib (CXB) inhibited the proliferation of LECs in vitro [94], Brookshire et al. [95] implanted IOLs into the eyes of experimental dogs after incubating IOLs in CXB solution, and this effectively reduced the postoperative inflammation. In the fourth week, the CXB-IOL group showed a better PCO prevention effect than the group treated by another COX-2 inhibitor, namely, bromfenac (BF) eye drops. However, the long-term (56 weeks) PCO prevention effect was significantly lower than the BF eye drops-treated group. Zhang et al. [96] pointed out that BF can inhibit the transforming growth factor-β2 (TGF-β2) through the signal-regulated kinase (ERK)/glycogen synthase kinase-3β (GSK-3β)/Snail signaling pathway. The team fabricated BF-loaded PLGA coatings on the haptic region of the IOL by ultrasonic spraying. BF-PLGA-IOL inhibited PCO formation in rabbit PCO models, which was more effective than the BF drops-treated group.

Antitumor drugs can generally be divided into cytotoxic drugs (such as 5-Fluorouracil (5-FU), DOX, and paclitaxel (PAC)) and non-cytotoxic drugs (such as gefitinib and erlotinib) [23]. However, the safety and efficacy of antineoplastic drugs used in the eyes require close attention. To reduce the toxicity of 5-FU, Huang et al. [97] prepared 5-FU-loaded CHI nanoparticles (NPs) and surface-modified IOLs with drug-loaded nanoparticles, which could sustainably release 5-FU after being implanted into the eye. The released 5-FU then inhibited LEC proliferation and promoted LEC apoptosis. Han et al. [98] prepared DOX-incorporated chitosan (CHI) NPs using a sodium tripolyphosphate (TPP) gel method. The positively charged CHI-TPP-DOX nanoparticles (CTDNPs) and the negatively charged heparin (HEP) self-assembled layer-by-layer on the IOL surface through electrostatic interaction to obtain the (HEP/CTDNP)_n_ multilayer-modified IOL. The LEC adhesion, proliferation, and migration on the modified IOL surface were significantly inhibited because of the surface hydrophilic modification combined with the drug-loading modification. This IOL effectively reduced the formation of PCO and the Soemmerring’s ring (SR). Similar studies focused on IOL modification by hydrophilic anti-biofouling coatings loaded with DOX or PAC have also confirmed that such multifunctional IOLs may have great potential in preventing PCO. This makes up for the shortcomings of simple hydrophilic anti-biofouling IOLs with poor long-term PCO prevention [99,100]. In another study, drug-eluting IOLs loaded with the EGF inhibitors, gefitinib or erlotinib, could effectively slow LEC proliferation in ex vivo models of the human lens capsular bag. Additionally, sustainable drug release showed no toxicity to corneal endothelial cells [101,102].

**Figure 8:**
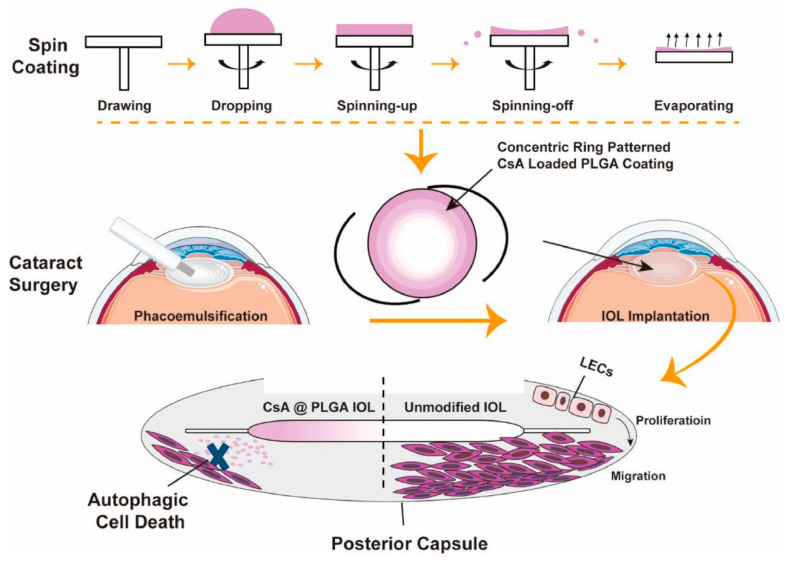
Schematic illustration of intraocular lens modified by centrifugally concentric ring-patterned drug-loaded PLGA coating for posterior capsular opacification prevention. Adapted with permission from Ref. [104]. Copyright 2022 Elsevier.

Immunosuppressants, such as cyclosporin A (CsA) and rapamycin (RAPA), also have the potential to inhibit cellular proliferation and PCO formation [23]. For example, the CsA-loaded PLGA (CsA@PLGA) coating-modified IOL prepared by Teng et al. [103] reduced the degree of PCO within six months after implantation in rabbit models. Using the spin-coating technique, Lu et al. [104] designed and fabricated a centrifugally concentric ring-patterned drug-loaded PLGA coating (Figure 8). The concentric ring morphology, drug loading, and release properties were investigated, and the spin-coating parameters were optimized. Then, they created a concentric coating with a thin center and a thick rim, which is especially suitable for the surface modification of the intraocular lens without affecting the optical quality of the IOL. The CsA@PLGA coating-modified IOLs significantly inhibited cell proliferation and induced cell death via the autophagy-mediated cell death pathway. In vivo intraocular implant results confirmed its inhibitory effect on PCO. In another study, the researchers compared the preventive effects of RAPA intraoperative capsular bag perfusion, postoperative RAPA eye drops, and PLGA coating-modified IOLs loaded with RAPA on PCO prevention [105]. The results showed that in the RAPA-PLGA-IOL group, the anterior RAPA concentration reached a peak at 4 days after surgery and continued to release RAPA for up to 8 weeks. Compared with the control group, only the RAPA-PLGA-IOL group can greatly reduce the degree of posterior capsular opacity, and there is no statistical difference in the degree of PCO among the other groups.

EMT usually demonstrates the loss of cell polarity and adhesion and the acquisition of migration and invasion capabilities. This is accompanied by the production of large amounts of extracellular matrix and the inhibition of cell apoptosis simultaneously. Furthermore, the cell migration and EMT process play essential roles in PCO pathogenesis. Methotrexate intraocular lenses prepared by supercritical impregnation reduce cellular fibrosis by inhibiting the EMT process [106]. Moreover, TGF-β is a key signaling molecule that induces LEC migration and the EMT process, which is usually used to construct EMT models [107]. The occurrence and development of PCO are expected to be inhibited by targeting TGF-β, TGF-β receptors, and their upstream and downstream signaling pathways. Sun et al. [108] successfully immobilized the anti-TGF-β2 antibody on the IOL surface by plasma pretreatment and layer-by-layer self-assembly technology. These IOLs maintained stability and immune activity for at least three months. Anti-TGF-β2 antibody-functionalized IOLs can directly target and bind TGF-β2 molecules after implantation in the capsular bag, thereby inhibiting the TGF-β2-induced LEC migration and EMT process after cataract surgery. As mentioned above, the molecular mechanism of bromfenac sodium targeting the TGF-β2 signaling pathway is used to inhibit the cell migration and EMT process of LECs [96].

**Table 3:** Summary of reviewed literature of drug-loaded IOLs for PCO prophylaxis with in vivo results.

Drug	Other Composition	Mechanism	Main Loading Method	Loading Dosage	IOL Type	Observation	Ref.
CXB	none	not verified in the article	immersion	unclear	acrylic IOL	56 weeks in dog model	[95]
BF	PLGA	inhibit cell migration and TGF-β2-induced EMT	ultrasonic spray technique	≈100 μg/IOL of BF	acrylic IOL (Wuxi Vision PRO)	four weeks in rabbit model	[96]
5-FU	CHI	inhibit cell proliferation and promote cell apoptosis	fluorine ion beam-aided activation and immersion	≈19.55 ± 1.31 mg/IOL of 5-FU	PMMA IOL (CJ55, Rafi Systems)	four weeks in rabbit model	[97]
DOX	CHI/TPP/HEP	inhibit cell adhesion, proliferation, and migration	ionic gelation, surficial activation, and layer-by-layer self-assembly	unclear	acrylic IOL (66Vision Tech.)	two months in rabbit model	[98]
DOX	PDA/MPC	inhibit cell adhesion, proliferation	self-polymerization and immersion	≈2.8 μg/IOL of DOX	acrylic IOL (66Vision Tech.)	six weeks in rabbit model	[99]
CsA	PLGA	inhibit cell proliferation and promote autophagy-mediated cell death	spin-coating technique	unclear	acrylic IOL (66Vision Tech.)	four weeks in rabbit model	[104]
RAPA	PLGA	not verified in the article	proprietary spray technique	unclear	PMMA IOL (Suzhou Medical Instrument)	three months in rabbit model	[105]

## 3. Biosecurity of IOLs in PCO Prophylaxis

Biosecurity issues of modified IOLs using different strategies for PCO prevention still need more attention and exploration. Researchers usually perform histopathology examinations of other eye tissues, including cornea, iris, and retina, to illustrate the biosecurity in IOLs. Nonetheless, they should also attach more importance to detections of intraocular pressure (IOP), corneal endothelial cells, and visual electrophysiology in vivo experiments. The IOP reflects the circulation situation of aqueous humor. Corneal endothelial cells are vital for keeping the corneal transparent. Additionally, visual electrophysiology (such as electroretinogram) demonstrates the retinal function.

Photothermal IOLs have nowadays been shown to exert a local heating effect and kill LECs in a limited field, as demonstrated by in vitro and in vivo experiments [76,78,83,84]. Since ROS generated in the PDT strategy has a short action distance (<20 nm) and limited lifetime (<40 ns), photodynamic IOLs were effective in killing adherent LECs on the surface and alleviated toxicity to other eye tissues [86,87]. Approaches of PTT and PDT for PCO prevention caused no significant changes in IOP and corneal endothelial cell count [85,86]. However, researchers have not achieved direct and accurate monitoring of intraocular temperature change. Additionally, new temperature monitoring technology is needed to help researchers optimize the assembling of photothermal materials and the use of illumination power. Nevertheless, retinal toxicity is a significant issue when applying photothermal or photodynamic IOLs with light irradiation. Mao et al. performed an electroretinogram test and found the retinal function stayed normal in the group of BP-DOX@IOL implantation with 808 nm laser irradiation treatment [85]. The drug-loaded IOLs are not yet used in clinical studies since it is vital to ensure that those pharmacological agents cause no effect on other ocular tissues. More specifically, toxicity to the corneal endothelium leads to the decompensation and opacification of the cornea [5,22,23]. Thus, we recommend that functional and structural analyses for evaluating the biocompatibility and biosecurity of modified IOLs be combined with specific treatments.

## 4. Conclusions and Future Perspectives

Scientists have developed different surface-modified IOLs using different strategies to disrupt PCO formation. Anti-biofouling IOLs can effectively resist the adhesion of proteins, cells, and even bacteria to reduce the intraocular inflammatory response after cataract surgery. Decreased LEC adhesion and migration on the IOL surface can reduce PCO incidence. Adhesion-enhanced IOLs can adhere more closely to the posterior capsule to form a sandwich structure of the IOL, monolayer LECs, and posterior capsule membrane, promoting LEC adhesion to inhibit LEC proliferation and differentiation, thereby preventing PCO. The surface micro-patterned IOL utilizes the specially designed surface topography to regulate LEC migration behavior to the center of the posterior capsule, effectively suppressing the opacity of the posterior capsule. Photothermal and photodynamic IOLs have introduced PTT and PDT therapy, which can safely and effectively remove residual LECs. Through controllable irradiation of specific wavelength light and distribution of photothermal materials or photosensitizers, PTT and PDT strategies will not damage other ocular tissues outside IOLs. Drug-loaded IOLs inhibit PCO development by loading various drugs that regulate the proliferation, migration, EMT, and other behaviors of residual LECs.

However, there are still some problems waiting to be solved in the future if we want to apply these new types of IOLs for PCO prevention in the clinical. The first one is biosecurity, discussed in Section 3. Researchers should perform rigorous and precise approaches to evaluating the use of laser or light irradiation and establish relatively uniform standards for the ophthalmic treatment of PCO. Certain issues, such as controlled and sustained release and ocular toxicity of drugs, require more in-depth research to ensure intraocular safety and the effectiveness of PCO prevention. The pharmaceuticals will ultimately be released from drug-loaded IOLs and lose their effect once the concentration in the lens capsule decreases below the effective concentration. Nonetheless, PTT and PDT strategies can be performed repeatedly under unlimited time constraints since the photothermal and photodynamic coatings can be stable on IOL surfaces. Additionally, anti-biofouling, enhance-adhesive, or micro-patterned modifications are also retained for a longer period of time than the drug-loading modification. Pharmacotherapy can be combined with other non-pharmaceutical strategies. On the other hand, methods for only regulating LECs behaviors, not killing, can be combined with methods for eliminating residual LECs. A single functional IOL may be less effective in long-term PCO prevention; thus, developing multifunctional IOLs united with different surface modifications can generate a more effective PCO prevention system. In vitro cell experiments and in vivo animal PCO model experiments have confirmed that many surface-modified IOLs have excellent biocompatibility and PCO prevention effects. However, there is still a lack of more reliable, clinically relevant research data, which requires further investigation.

The surface-modified intraocular lenses with different functions require the cross-integration of multidisciplinary theoretical knowledge, such as materials science, medicine, pharmacology, and cell biology. They have broad clinical application prospects in the future prevention and treatment of PCO.

## Data Availability

Not applicable.
